# Cognitive and Emotional Effect of a Multi-species Probiotic Containing *Lactobacillus rhamnosus* and *Bifidobacterium lactis* in Healthy Older Adults: A Double‐Blind Randomized Placebo‐Controlled Crossover Trial

**DOI:** 10.1007/s12602-024-10315-2

**Published:** 2024-06-27

**Authors:** Cristofer Ruiz-Gonzalez, Diana Cardona, Lola Rueda-Ruzafa, Miguel Rodriguez-Arrastia, Carmen Ropero-Padilla, Pablo Roman

**Affiliations:** 1 Torrecárdenas University Hospital, Almeria, Andalusia 04009 Spain; 2 Research Group CTS-1114 Advances and Innovation in Health, University of Almeria, Almeria, Andalusia 04120 Spain; 3 Department of Nursing Science, Physiotherapy and Medicine, Faculty of Health Sciences, University of Almeria, Almeria, Andalusia 04120 Spain; 4 Health Research Center CEINSA, University of Almeria, Almeria, Andalusia 04120 Spain; 5 ScienceFlows, Universitat de València, Valencia, 46010 Spain

**Keywords:** Affective, Aging, Gut microbiota, Mental, Microbiota modulation

## Abstract

As the population ages, cognitive decline becomes more common. Strategies targeting the gut-brain axis using probiotics are emerging to achieve improvements in neuropsychiatric and neurological disorders. However, the beneficial role of probiotics on brain function in healthy older adults remains unclear. Our aim was to evaluate a multi-species probiotic formulation as a therapeutic approach to reduce emotional and cognitive decline associated with aging in healthy adults. A randomized double-blind placebo-controlled crossover trial was conducted. The study involved a 10-week intervention where participants consumed the assigned probiotic product daily, followed by a 4-week washout period before the second condition started. Cognitive function was assessed using the Mini-Mental State Examination (MMSE) and the Psychological Experiments Construction Language Test Battery. At the emotional level, the Beck Depression Inventory (BDI) and the State-Trait Anxiety Inventory (STAI) were used. Thirty-three participants, recruited between July 2020 and April 2022, ingested a multispecies probiotic (*Lactobacillus rhamnosus* and *Bifidobacterium lactis*). After the intervention, noticeable enhancements were observed in cognitive function (mean difference 1.90, 95% CI 1.09 to 2.70, *p* < 0.005), memory (mean difference 4.60, 95% CI 2.91 to 6.29, *p* < 0.005) by MMSE and digit task, and depressive symptoms (mean difference 4.09, 95% CI 1.70 to 6.48, *p* < 0.005) by BDI. Furthermore, there were significant improvements observed in planning and problem-solving skills, selective attention, cognitive flexibility, impulsivity, and inhibitory ability. Probiotics administration improved cognitive and emotional function in older adults. Limited research supports this, requiring more scientific evidence for probiotics as an effective therapy for cognitive decline. This study has been prospectively registered at ClinicalTrials.gov (NCT04828421; 2020/July/17).

## Introduction

Ageing is a natural biological progression marked by a gradual deterioration of the mechanisms responsible for maintaining the organism’s homeostasis [1, 2]. The central nervous system undergoes a number of changes with ageing, such as brain atrophy, increased oxidative stress, and the generation of a pro-inflammatory state, as well as vascular deterioration leading to cognitive impairment and mood disorders [3, 4]. At the epidemiological level, population growth leads to an increase in the number of older people with a consequent increase in the occurrence of age-related health problems [5].

In this context, the search for new approaches to treat cognitive and emotional decline in old age, such as the modulation of gut microbiota (GM) and its relationship to ageing, is beginning to attract considerable scientific interest. GM is an ecosystem comprised of live microorganisms that inhabit the human gastrointestinal tract and is essential to promote nutrient absorption, protect the host from pathogen invasion, produce metabolites related to energy homeostasis, and play an important role in immune system modulation [6, 7]. In recent years, the emergence of the concept of gut-brain axis (GBA) [8] as a bidirectional communication pathway between the gastrointestinal tract and the brain has made it possible to link disorders of the GM with neurodegenerative pathologies such as Alzheimer’s (AD) and Parkinson’s disease (PD) or mood disorders including anxiety and depression [9–11]. To date, it has been proposed that changes in GM composition, decreases in *Firmicutes*/*Bacteroidetes* ratio, or increases in *Clostridium* spp. metabolites would trigger a neuroinflammatory environment, mitochondrial dysfunction, or oxidative stress promoting neuronal impairment [12, 13].

A wide variety of dietary strategies including the consumption of probiotics, prebiotics, or symbiotics have been investigated to achieve beneficial effects in patients with gut dysbiosis associated with neurological problems [14, 15]. The impact of probiotics on cognitive function through GBA has been studied in multiple conditions such as dementia, cognitive impairment, and affective disorders such as anxiety and depression. Thus, the consumption of probiotics of the genera *Bifidobacterium* and *Lactobacillus* has been associated with improved scores for general cognitive function, inflammatory status, and brain neurotrophin levels in patients with AD [16–18]. Concerning cognitive impairment, an improvement in memory has been demonstrated after administration of *Bifidobacterium breve* A1 [19], as well as in learning and verbal fluency after consumption of *Limosilactobacillus fermentum* and *Lactobacillus plantarum* C29 [20–22]. In patients with anxiety and depression, the probiotic combination of *Lactobacillus helveticus* R0052 and *Bifidobacterium longum* R0175 reduced symptoms of the disease, improved problem-solving ability, and reduced urinary-free cortisol levels [23]. In addition to the improvement in cognitive function, imaging tests have shown anatomical and functional changes in different brain areas involved in the pathophysiology of affective disorders following probiotic consumption [24]. However, most of the results are derived from individuals with multiple pathologies so it is crucial to assess whether such findings can be transferred to the healthy population, with a special focus on the elderly [25]. Therefore, the aim of this study is to evaluate the efficacy of a multi-species probiotic formulation as a therapeutic strategy to attenuate the emotional and cognitive decline associated with ageing in healthy adults.

## Methods

### Study Design

The study was a randomized, double-blind, placebo-controlled crossover trial conducted between July 2020 and April 2022 and designed according to the recommendations of the Consolidated Standards of Reporting Trials guidelines for randomized trials [26]. Participants were randomly assigned to one of the following two conditions: Placebo-Probiotic or Probiotic-Placebo. The study included a 10-week intervention period and a 4-week washout period between the two conditions. During the intervention period, participants consumed the assigned products once daily for 10 consecutive weeks. During the washout period, eligible participants were instructed to maintain the same dietary habit followed since the beginning of the evaluation, with the absence of intervention being the only distinctive feature. Assessments were conducted in three phases: at baseline, post 10-week intervention (first condition), and at the end of the study (after 10-week intervention of the second condition and 4 weeks of washout period). This work was prospectively registered on ClinicalTrials.gov; further details are provided in the previously published study protocol [27].

### Participants

The study sample consisted of 33 participants and was considered adequate according to the sample size calculations specified in the protocol [27]. Subjects were selected according to the following inclusion criteria (i) being 55 years of age or older, (ii) voluntarily agreeing to participate in the study in accordance with the Declaration of Helsinki, and (iii) not being involved in another study that could interfere with the results. Conversely, participants were excluded if they (i) had a severe mental illness, (ii) had a score below 10 on the Mini-Mental State Examination (MMSE), (iii) were taking medications affecting cognition, the microbiome, or gastrointestinal motility, or (iv) had another severe illness.

### Study Interventions

Participants received either placebo or probiotics. Participants were asked to consume a capsule containing a multi-species probiotic (3.3 billion CFU *Lactobacillus rhamnosus* and *Bifidobacterium lactis*) after breakfast. Regarding placebo, each capsule contained potato starch. Both interventions were indistinguishable by packaging, color, taste, and smell. Both products were stable at room temperature and were packaged in a blister pack of 15 oral capsules. To assess compliance, participants regularly brought blister packs of consumed capsules to the designated location. Alternatively, if not feasible, follow-up was conducted via video conferencing.

### Randomization and Masking

Eligible participants were distributed in a 1:1 ratio to the two groups (probiotic/placebo or placebo/probiotic), according to a computer-generated random sequence by the study coordinator, who was not involved in the trial. Sachets were pre-packaged according to the randomization code and an independent researcher from the study dispensed them to the participants. No member of the research team knew the assigned sequence until the end of the work and the blocking of the database. The study was unmasked after all statistical analyses were completed.

### Outcomes

For each of the participants, an assessment of socio-demographic, primary (cognitive and emotional state), secondary, and confounding variables was carried out (Table 1). Specific data on the variables studied and the instruments used can be found in a study we recently published [27]. Briefly, an ad hoc questionnaire was used to assess socio-demographic variables, the STAI and BDI-I to evaluate emotional status, the MMSE and PEBL battery to assess cognitive functions, and the PSQI and Bristol scale to analyze secondary variables.

**Table 1:** Study variables and data collection tools

**Variables**		**Tool**		**Data/function**
Sociodemographic		Ad hoc* questionnaire*		Age, sex
				Marital status
				Employment status
				Educational level
				Digestive problems
				Tobacco use
				Elimination pattern
				Digestive diseases
				Nutritional supplements/components use (including consumption of probiotics, prebiotics or any GM modulator, caffeine, and other stimulants)
				Regular medication
				Antibiotic use in the previous month
Primary variables	Cognitive	*MMSE*		General cognitive status: orientation, fixation, concentration and calculation, memory and language, and construction
		*PEBL*	*Tower of London*	Planning ability
			*Digit and Corsi Task*	Working memory
			*Wisconsin Card Sorting test*	Problem‐solving ability
			*Stroop task*	Selective attention, cognitive flexibility, and response inhibition
			*Trail Making test*	Visual attention and the ability to switch tasks
			*Iowa Gambling task*	Choice impulsivity
			*Go/No‐Go task*	Motor impulsivity or inhibitory response control
	Emotional	*BDI-I*		Depression
		*STAI*		State and trait anxiety
Secondary variables		*Bristol scale*		The type and consistency of bowel movements
		*PSQI*		Sleep quality
Potential confounding variables		*“24‐hour recall” questionnaire*		Dietary habits
		*AUDIT*		Alcohol use
		*OMRON BF511 bioimpedance meter*		BMI, body and visceral fat
		*IPAQ*		Physical activity
		*PSQ*		Stress
		*GSRS*		

*AUDIT* Alcohol Use Disorders Identification Test, *BDI-I* Beck depression inventory, *BMI* Body Mass Index, *GSRS* Gastrointestinal Symptom Rating Scale, *GM* Gut Microbiota, *IPAQ* International Physical Activity Questionnaire, *MMSE* Mini-Mental State Examination, *PEBL* Psychology Experiment Building Language Test Battery, *PSQ* Perceived Stress Questionnaire, *PSQI* Pittsburgh Sleep Quality Index Questionnaire, *STAI* State and Trait Anxiety Inventory

### Procedures

**Fig. 1:**
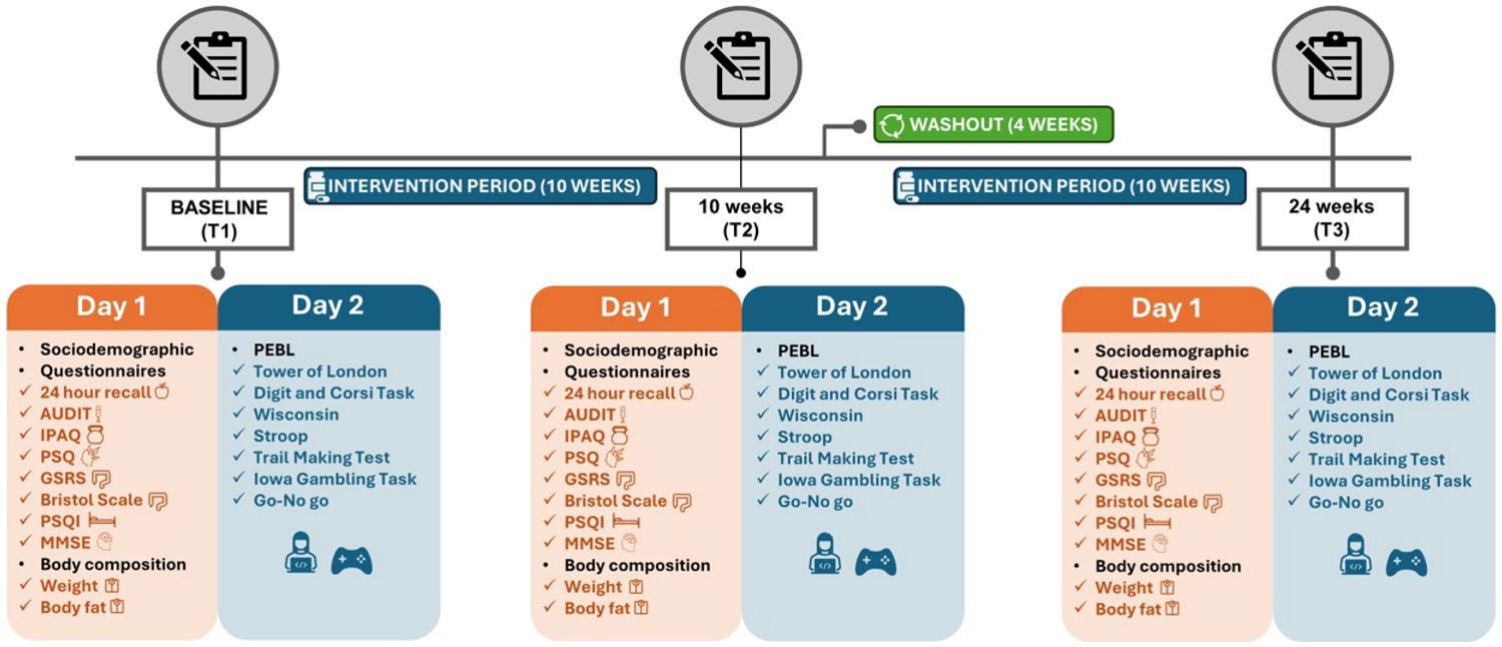
Timeline of testing sessions

Study participants were recruited through a variety of channels (brochures, e-mail, senior university, and others). Once recruitment was completed, those who expressed interest in participating were assessed to determine whether they met the required eligibility criteria and, if so, signed a written informed consent form. Participants were assessed at the start of the study, administered one condition (placebo/probiotic) for 10 weeks, and then switched to the other condition (probiotic/placebo) for a further 10 weeks (Fig. 1). Participants had a 4-week washout period between conditions. Participants were instructed to take the probiotic or placebo at home and regular contact was maintained to verify that scheduled procedures were being followed properly throughout the intervention period. Treatment was administered in a staggered manner, with each participant receiving blister packs of capsules twice for each condition, to ensure adherence to the medication and to quantify the number of capsules used. Data collection was conducted in two phases: first, paper questionnaires were completed for the assessment of sociodemographic variables and emotional state and the next day a computer was used to complete the virtual tests for the assessment of cognitive function. The evaluative tests included an initial simulation for participants to familiarize themselves with the exercise in each phase of the study.

### Statistical Analysis

Continuous variables were expressed as mean and standard deviation (SD) or median, range (maximum and minimum values) according to their distribution, and categorical variables were expressed using a table of frequencies and percentages. Subsequently, the Kolmogorov–Smirnov test was applied to determine the normality of the data. For both means and proportions, 95% confidence intervals were obtained. Repeated measures analysis of variance and pairwise tests using Fisher’s post hoc least significant difference or non-parametric equivalents (Friedman and Wilcoxon respectively) were used to compare results in both conditions. Categorical variables were compared with the *χ*2 test or Fisher’s exact test. Finally, effect size measures (*η*2) were generated for each analysis reaching statistical significance, providing information on the variability in results that can be attributed to our experimental manipulations. All statistical analyses were conducted using IBM SPSS Statistics for Windows, version 26 (IBM Corp).

### Ethical Considerations

This study was approved by the Ethics Committee of from University of Almería (UALBIO2020-001) and has complied in all its phases with the international ethical requirements of the Declaration of Helsinki, always ensuring the confidentiality of the participants as well as their participation and voluntary follow-up in the research. Participants were not compensated for their participation in the study.

## Results

### Subjects

**Fig. 2:**
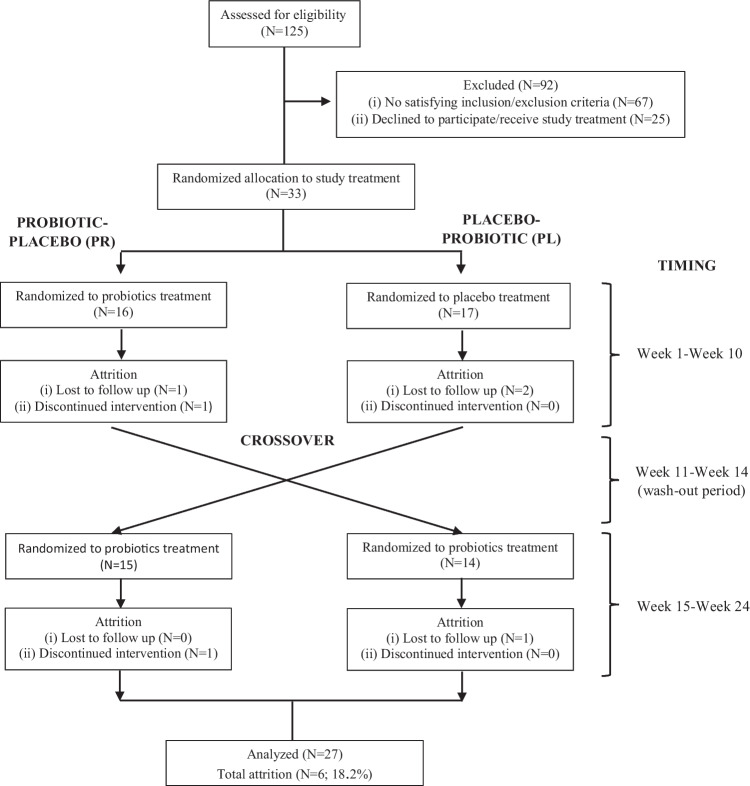
Flow diagram of all participants throughout the study stages

Thirty-three individuals were selected after applying the criteria from a pool of 125 participants. The individuals were recruited between July 1, 2020, and April 25, 2022. Figure 2 shows the CONSORT flowchart of the participants in the study. Six subjects did not complete the first (*n* = 4) or second (*n* = 2) arm of the intervention and were considered in the intention-to-treat analysis (ITT) (Fig. 2; study attrition rate, 18.2%). No side effects were experienced. Table 2 presents the socio-demographic and baseline data of the participants (*n* = 27) who completed the two 10-week intervention periods. The mean age was 66.22 years and 18/30 were women. In relation to gastrointestinal function, 44% of the included older adults had digestive problems, with flatulence, bloating, belching, and loose stools being the most common symptoms. In terms of general cognitive function, 85.2% of participants showed no cognitive impairment and 14.8% showed mild cognitive impairment prior to baseline.

**Table 2:** Sociodemographic variables

**Variable**	*N* = 33
**Age (mean [SD])**	65.94 (6.17)
**Sex (*n* [%])**	
Male	10 (30)
Female	23 (69)
**Marital status (*n* [%])**	
Single	2 (6)
Married	24 (73)
Divorced	3 (9)
Widowed	4 (12)
**Employment status (*n* [%])**	
Unemployed	4 (12)
Employed	8 (24)
Retired	21 (63.6)
**Educational level (*n* [%])**	
Elementary school	12 (36)
Middle school	11 (33)
High school	3 (9)
University studies	7 (21)
**Chronic diseases (*n* [%])**	
Yes	26 (79)
No	7 (21)
**Digestive problems (n [%])**	
Yes	13 (39)
No	20 (61)
**Tobacco use (*n* [%])**	
Yes	1 (3)
No	32 (97)
**Antibiotic use in the last 3 months (*n* [%])**	
Yes	7 (21)
No	26 (79)
**Regular medication use (*n* [%])**	
Yes	27 (82)
No	6 (18)
**BMI** (mean [SD])	27.5 (3.12)
**Body fat** (mean [SD])	35.3 (8.83)
**Visceral fat** (mean [SD])	10.7 (3.64)
**Gastrointestinal Symptom Rating Scale (GSRS)**^**1**^	
Total Score (mean [SD])	12.15 (7.15)
Reflux (mean [SD])	2.2 (1.27)
Abdominal pain (mean [SD])	2.0 (1.15)
Indigestion (mean [SD])	3.0 (1.65)
Diarrhea (mean [SD])	2.3 (1.41)
Constipation (mean [SD])	2.6 (1.65)
**Perceived Stress Questionnaire (PSQ)**^**2**^	
Last year (mean [SD])	63.5 (6.75)
Last month (mean [SD])	64.1 (6.75)
**Alcohol Use Disorders Identification Test (AUDIT)**^**3**^	0.8 (1.56)
Total score (mean [SD])	
**International Physical Activity Questionnaire (IPAQ)**^**4**^	
Total score (mean [SD])	2217.2 (378.5)
**Mini-Mental State Examination (MMSE)**^**5**^	
Total score (mean ± [SD])	32.6 ± 0.4 (2.30)
Total score (range)	

Data are expressed as means ± standard deviation (SD) or as number of cases (%). The GSRS^1^ is a 15-item questionnaire that assesses gastrointestinal symptoms on a scale from 1 (no discomfort at all) to 7 (very severe discomfort) for five syndromes which include abdominal pain (abdominal pain, hunger pains and nausea), diarrhea (diarrhea, loose stools and urgent need for defecation), constipation (constipation, hard stools and feeling of incomplete evacuation), indigestion (borborygmus, abdominal distension, eructation and increased flatus), and reflux (heartburn and acid regurgitation). The score for each syndrome has been calculated by taking the mean of their symptom values. Total score indicates the sum of the mean scores obtained for each syndrome, with higher scores indicating greater severity of symptoms. The PSQ^2^ is a 30-item questionnaire that assesses factors that influence stress on a scale from 1 (“almost never”) to 4 (“almost always”) for six factors which include Tension-Instability-Fatigue, Social Acceptance of Conflict, Energy and Fun, Overload, Satisfaction for Self-Realization, and Fear. It is evaluated in two different periods (recently and in the last 2 years). The AUDIT^3^ is a 10-item questionnaire that measures recent alcohol use, alcohol dependence symptoms, and alcohol-related problems on a scale from 0 (“never”) to 4 (“daily”). The total score is calculated by summing the scores of each individual question. The IPAQ^4^ is a 7-item questionnaire that measures physical activity levels by obtaining information on time spent walking, doing moderate/vigorous activities, and sitting during the past 7 days. The total score was obtained by summing the scores obtained in each category multiplied by the Metabolic Equivalent of Task (MET). The MMSE^5^ is a questionnaire that assesses overall cognitive status in five areas: orientation, fixation, concentration and calculation, memory and language, and construction. The total score is obtained by summing the scores obtained in each category

### MMSE

The data revealed differences between the groups in the MMSE test (*p* < 0.0001). Significant differences were found between the probiotic condition with the baseline (mean difference 1.90, 95% CI 1.09 to 2.70, *p* < 0.005) and the placebo condition (mean difference 1.80, 95% CI 1.18 to 2.42, *p* < 0.005). Changes were observed in orientation, concentration/calculation, and memory, with the latter dimension showing a mean score improvement of + 1.1 for the probiotic condition compared to the placebo condition (Table 3).

**Table 3:** Cognitive status variables

**Cognitive status**	**Baseline (BS)**	**Placebo (PL)**	**Probiotic (PR)**	***F*** **(PP analysis)**	***p*-level**	***p*-level ITT**	***η*2**_**π**_
MMSE							
Total score (SD)	32.6 (2.07)	32.7 (1.54)	34.5 (0.52)^a,b^	42.975	< 0.0001	< 0.0001	0.623
Orientation (SD)	9.8 (0.51)	9.8 (0.52)	10.0 (0.00)	3.25	0.032	0.02	0.111
Fixation (SD)	3.0 (0.01)	3.0 (0.00)	3.0 (0.01)	0.132	1	1	0.053
Concentration/calculation (SD)	7.2 (0.89)	7.3 (1.21)	7.8 (0.52)^a^	7	0.001	0.001	0.212
Memory (SD)	1.6 (1.12)	1.6 (1.04)	2.7 (0.52)^a,b^	32.579	< 0.0001	< 0.0001	0.556
Language/construction (SD)	11.0 (0.01)	10.9 (0.52)	11.0 (0.00)	2.08	0.135	0.135	0.074
Digit task							
Total score (SD)	13.1 (2.60)	12.7 (3.12)	17.7 (3.64)^a,b^	119.248	< 0.0001	< 0.0001	0.821
Forward (SD)	8.5 (2.08)	8.5 (2.09)	10.7 (2.11)^a,b^	66.942	< 0.0001	< 0.0001	0.72
Reverse (SD)	4.7 (1.56)	4.3 (1.04)	7.0 (2.08)^a,b^	44.46	< 0.0001	< 0.0001	0.631
Tower of London							
Total time (SD)	18.6 (3.64)	18.1 (4.16)	15.7 (4.15)^a,b^	10.666	0.001	< 0.0001	0.291
Successful rounds (SD)	12.6 (3.17)	14.4 (3.13)^a^	20.0 (3.12)^a,b^	120.826	< 0.0001	< 0.0001	0.766
Corsi task							
Total score (SD)	30.2 (12.47)	28.6 (11.43)	45.6 (15.06)^a,b^	52.95	< 0.0001	< 0.0001	0.571
Correct answers (SD)	6.3 (1.48)	6.3 (1.56)	8.0 (1.30)^a,b^	63.561	< 0.0001	< 0.0001	0.629
Memory capacity (SD)	4.1 ± (0.52)	4.1 (1.04)	5.0 (0.51)^a,b^	62.547	< 0.0001	< 0.0001	0.629
Wisconsin Card Sorting test							
Correct response (SD)	48.3 (5.72)	52.6 (3.12)^a^	56.9 (2.08)^a,b^	53.525	< 0.0001	< 0.0001	0.597
Categories achieved (SD)	3.3 (1.04)	3.7 (1.04)	4.7 (0.52)^a,b^	26.066	< 0.0001	< 0.0001	0.425
Perseverative errors (SD)	9.1 (3.64)	8.7 (2.60)	5.7 (1.04)^a,b^	22.843	< 0.0001	< 0.0001	0.38
Non-perseverative errors (SD)	6.6 (6.24)	2.7 (3.12)	1.5 (1.56)^a,b^	16.329	< 0.0001	< 0.0001	0.331
Set maintenance errors (SD)	0.6 (1.05)	0.8 (1.04)	0.3 (0.52)	3.76	0.148	0.027	0.085
Stroop task							
Congruent reaction time (SD)	24.0 (7.27)	21.3 (6.24)^a^	19.1 (4.16)^a,b^	12.874	< 0.0001	< 0.0001	0.33
Incongruent reaction time (SD)	26.6 (7.79)	20.8 (4.68)	20.8 (4.67)^a,b^	22.041	< 0.0001	< 0.0001	0.496
Congruent errors (SD)	0.6 (1.12)	1.0 (1.27)	0.4 (0.79)^b^	10.517	0.002	< 0.0001	0.212
Incongruent errors (SD)	4.7 (3.12)	5.1 (5.20)	1.9 (0.52)^a,b^	29.405	< 0.0001	< 0.0001	0.447
Trail making test							
Single Targets (SD)	12.3 (3.08)	13.3 (3.12)	17.4 (4.16)^a,b^	39.248	< 0.0001	< 0.0001	0.502
Single reaction time (SD)	20.1 (0.00)	20.2 (0.52)	20.2 (0.01)	3.195	0.708	0.68	0.063
Single clicks (SD)	12.9 (2.60)	13.8 (3.12)	17.8 (4.16)^a,b^	37.337	< 0.0001	< 0.0001	0.489
Single overclicks (SD)	2.1 (2.36)	1.8 (2.60)	1.5 (2.88)	0.812	0.546	0.618	0.021
Switch targets (SD)	9.1 (2.08)	9.9 (2.09)	13.0 (2.60)^a,b^	42.869	< 0.0001	< 0.0001	0.528
Switch reaction time (SD)	20.1 (0.00)	20.1 (0.01)	20.1 (0.02)	0.973	0.470	0.352	0.024
Switch clicks (SD)	10.0 (1.98)	10.6 (2.25)	13.2 (2.30)^a,b^	33.752	< 0.0001	< 0.0001	0.466
Switch overclicks (SD)	3.8 (3.12)	3.2 (4.67)	1.2 (1.56)^a^	7.146	0.014	0.002	0.155
R/D targets (SD)	0.8 (0.15)	0.8 (0.13)	0.8 (0.09)	0.128	0.994	0.153	0.00
R/D reaction time (SD)	1.0 (0.003)	1.0 (0.004)	1.0 (0.004)	5.708	0.352	0.02	0.043
R/D clicks (SD)	− 3.0 (2.13)	− 3.2 (2.08)	− 4.5 (2.09)^a,b^	9.951	0.004	0.001	0.216
R/D overclicks (SD)	1.6 (4.67)	1.5 (3.64)	− 0.3 (2.08)	4.871	0.100	0.017	0.091
Iowa Gambling Task (IGT)							
IGT index (SD)	− 6.2 (16.11)	− 13.9 (17.15)	34.3 (10.91)^a,b^	132.204	< 0.0001	< 0.0001	0.778
Disadvantageous A (SD)	21.2 (5.20)	25.8 (4.16)	15.2 (3.64)^a,b^	67.97	< 0.0001	< 0.0001	0.646
Disadvantageous B (SD)	31.4 (8.31)	31.2 (8.83)	17.7 (3.12)^a,b^	47.569	< 0.0001	< 0.0001	0.561
Advantageous C (SD)	16.(6.24)	17.2 (5.20)	17.5 (4.16)	0.475	0.829	0.699	0.01
Advantageous D (SD)	30.0 (7.79)	25.9 (8.31)	49.6 (7.79)^a,b^	104.26	< 0.0001	< 0.0001	0.733
Go/No‐Go task							
Response time-Go (SD)	0.5 (0.53)	0.5 (0.52)	0.5 (0.51)	2.177	0.327	0.075	0.041
Response time-No-Go (SD)	0.6 (0.49)	0.6 (0.52)	0.5 (0.53)^b^	8.139	0.033	< 0.0001	0.158
Percentage of omission errors-Go (SD)	4.6 (7.27)	6.6 (10.91)	1.55 (2.08)^a,b^	6.666	< 0.0001	< 0.0001	0.148
Percentage of commission errors-Go (SD)	3.4 (4.16)	6.8 (8.83)	0.9 (2.60)^b^	13.795	< 0.0001	< 0.0001	0.377
Percentage of omission errors-No-Go (SD)	23.9 (9.35)	22.5 (14.03)	11.5 (8.31)^a,b^	21.539	< 0.0001	< 0.0001	0.287
Percentage of commission errors-No-Go (SD)	1.6 (1.56)	1.9 (1.56)	1.0 (1.04)	4.052	0.112	0.045	0.097

Repeated-measures analyses of variance (ANOVA) was performed. The different treatments (probiotic or placebo) were entered as within-subject factors. Data are expressed as means ± standard deviation (SD) for the baseline, probiotic, and placebo group

*ITT* intention to treat, *PP* per protocol, *R* round, *R/D* ratio/difference, *η*^*2*^_*p*_ partial eta squared

^a^*p* < 0.05 difference from baseline value; ^b^*p* < 0.05 difference from placebo value

### Digit Task

For each participant, the memory ability score was calculated based on the longest sequence that was correctly recalled, forwards, and backwards. The digit task showed differences between groups for the total score (*p* < 0.0001). The post-hoc test revealed a significant difference when comparing the score obtained in the probiotic group with the baseline (mean difference 4.60, 95% CI 2.91 to 6.29, *p* < 0.005) and placebo (mean difference 5.00, 95% CI 3.19 to 6.81, *p* < 0.005). The improvement in test score was observed both in the direct order (baseline/placebo-probiotic: + 2.2) and in the reverse order (baseline-probiotic: + 2.3; placebo-probiotic: + 2.7) (Table 3).

### Tower of London

Differences between groups were observed for total time (*p* < 0.0001) and successful rounds (*p* = 0.001). In this regard, the post-hoc test indicated a significant reduction in the first variable and a significant increase in the second measure, respectively, in the probiotic condition compared to the baseline conditions (mean difference 2.90; 95% CI: 0.82 to 4.98; *p* < 0.005; mean difference 7.40; 95% CI: 5.74 to 9.06; *p* < 0.005) and placebo (mean difference 2.40; 95% CI: 0.18 to 4.62; *p* < 0.005; mean difference 5.60; 95% CI: 3.94 to 7.26; *p* < 0.005) (Table 3).

### Corsi Task

The results suggested group variations for total score obtained in the Corsi task (*p* < 0.0001). Post-hoc analysis showed significant differences when comparing the total score on the probiotic with the baseline (mean difference 15.40, 95% CI 8.02 to 22.78, *p* < 0.005) and the placebo (mean difference 17.00, 95% CI 9.87 to 24.13, *p* < 0.005). In addition, there was a significant improvement in memory capacity of + 0.9 in the probiotic compared to each of the other conditions (Table 3).

### Wisconsin Card Sorting Test

The analysis found differences between the groups for correct answers, categories achieved, and perseverative and non-perseverative errors (*p* < 0.0001). The post-hoc test showed that this difference was significant when comparing probiotic with baseline and placebo. No significant differences were found for perseverative responses (Table 3).

### Stroop Task

Mean measures of RT and errors obtained by participants’ trials were compared to estimate Stroop effects. Group differences were observed for congruent and incongruent errors (*p* = 0.002; *p* < 0.0001) and reaction time in both conditions (*p* < 0.0001). According to the post-hoc analysis, a significant reduction of incongruent errors and reaction time (congruent and incongruent) was revealed in the probiotic compared to the baseline and placebo (Table 3).

### Trail Making Test

In the simple and switched conditions, the data showed differences between the groups in relation to targets and clicks (*p* < 0.0001). This difference was statistically significant when comparing the probiotic group with the placebo and baseline groups, according to the post-hoc analysis. No changes were observed regarding reaction time (Table 3).

### Iowa Gambling Task

There were group differences according to the IGT index obtained in the task (*p* = 0.0001). The post-hoc test showed significant changes in this measure when comparing the probiotic group with the baseline (mean difference 40.50, 95% CI 33.16 to 47.84, *p* < 0.005) and placebo (mean difference 48.20, 95% CI 40.53 to 55.87, *p* < 0.005) (Table 3).

### Go/No‐Go Task

The percentage of errors in the Go conditions (errors of omission) and in the No-Go conditions (errors of commission) and the mean of the medians of the randomized trials obtained in the Go trials were analyzed (Table 3) considering both groups. Significant differences were found between the groups for the percentages of omission errors, commission errors in the Go condition (*p* < 0.0001), response time (*p* < 0.0001), and the percentage of omission errors in the No-Go condition (*p* = 0.033). The percentage of omission errors in the Go and No-Go conditions varied significantly in the probiotic group compared to baseline (mean difference 3.05, 95% CI 0.20 to 5.90, *p* < 0.005; mean difference 12.40, 95% CI 7.68 to 17.12, *p* < 0.005) and placebo (mean difference 5.05, 95% CI 0.86 to 9.24, *p* < 0.005; mean difference 11.00, 95% CI 4.85 to 17.15, *p* < 0.005), indicating an improvement for attention and impulsivity. The percentage of commission errors in the Go condition and the response time in the No-Go condition only showed significant differences between the probiotic and placebo groups (mean difference 5.90, 95% CI 2.43 to 9.37, *p* < 0.005; mean difference 0.10, 95% CI 0.18 to 0.28, *p* < 0.005).

### Emotional Status

The Beck Depression Inventory (BDI) total score showed a significant change between groups (*p* < 0.0001). According to the post-hoc test, the mean BDI score decreased significantly in the probiotic group compared to the baseline (mean difference 4.09, 95% CI 1.70 to 6.48, *p* < 0.005) and placebo (mean difference 4.29, 95% CI 1.90 to 6.68, *p* < 0.005) over 10 weeks (Table 4).

**Table 4:** Emotional status variables

**Emotional status**	**Baseline**	**Placebo**	**Probiotic**	***F*** **(PP analysis)**	***p*-level**	***p*-level ITT**	***η*2**_**π**_
**Beck depression inventory (BDI)**							
Total score (SD)	10.2 (5.05)	10.4 (5.12)	6.11 (3.62)^a,b^	101.54	< 0.0001	< 0.0001	0.796
**STAI**							
State anxiety (SD)	20.6 (7.88)	21.8 (7.23)	25.0 (2.86)	577.422	0.070	0.016	0.236
Trait anxiety (SD)	23.26 (7.65)	24.1(7.09)	26.0 (4.01)	4.295	0.041	0.046	0.142

Data are expressed as means ± standard deviation (SD) for the baseline, probiotic, and placebo group

*ITT* intention to treat, *PP* per protocol, *η*^*2*^_*p*_ partial eta squared

^a^*p* < 0.05 difference from baseline value; ^b^*p* < 0.05 difference from placebo value

There were group differences according to the global score obtained for both state anxiety (*p* = 0.070) and trait anxiety (*p* = 0.041) using the State-Trait Anxiety Inventory (STAI). However, no significant differences were found between the groups (Table 4).

### Bristol Scale

No statistically significant differences were found between the groups in stool consistency and stool frequency before and after treatment (Table 5).

**Table 5:** Secondary variables

**Secondary variables**	**Baseline**	**Placebo**	**Probiotic**	***F*** **(PP analysis)**	***p*-level**	***p*-level ITT**	***η*2**_**π**_
**Bristol Scale**							
Last month (SD)	4.3 (1.68)	4.4 (1.42)	4.3 (0.91)	0.067	0.979	0.984	0.003
Last week (SD)	4.4 (1.42)	4.3 (1.44)	4.2 (0.52)	0.704	0.220	0.428	0.026
Last stool (SD)	4.3 (1.46)	4.2 (1.42)	4.0 (0.44)	0.610	0.397	0.573	0.023
Bowel movement frequency (SD)	2.7 (0.78)	2.8 (0.64)	2.9 (0.27)	2.614	0.097	0.017	0.091
**Pittsburgh Sleep Quality Index Questionnaire**							
Total score (SD)	9.4 (3.88)	9.9 (4.00)	5.6 (2.34)^a,b^	67.925	< 0.0001	< 0.0001	0.723
Subjective sleep quality (SD)	1.5 (0.70)	1.5 (0.64)	1.0 (0.62)^a,b^	24.400	< 0.0001	< 0.0001	0.484
Sleep latency (SD)	1.5 (1.05)	1.7 (0.99)	1.0 (0.65)^a,b^	21.864	< 0.0001	< 0.0001	0.457
Sleep duration (SD)	1.3 (1.00)	1.5 (1.01)	0.6 (0.74)^a,b^	30.502	< 0.0001	< 0.0001	0.540
Stay in bed (SD)	8.1 (1.14)	8.1 (0.91)	8.5 (1.01)	5.170	0.003	0.042	0.166
Sleep efficiency (SD)	1.6 (1.28)	1.8 (1.21)	1.0 (1.01)^b^	7.744	0.001	0.001	0.229
Sleep disturbance (SD)	1.6 (0.57)	1.6 (0.57)	1.1 (0.52)^a,b^	28.000	< 0.0001	< 0.0001	0.519
Hypnotic use (SD)	1.2 (1.23)	1.1 (1.19)	0.6 (0.79)	8.714	< 0.0001	0.001	0.251
Diurnal dysfunction (SD)	0.7 (0.59)	0.7 (0.56)	0.2 (0.40)^a,b^	19.900	< 0.0001	< 0.0001	0.434

Data are expressed as means ± standard deviation (SD) for the baseline, probiotic, and placebo group

*ITT* intention to treat, *PP* per protocol, *η*^*2*^_*p*_ partial eta squared

^a^*p* < 0.05 difference from baseline value; ^b^*p* < 0.05 difference from placebo value

### Pittsburgh Sleep Quality Index Questionnaire (PSQI)

Sleep quality was significantly improved in the probiotic group compared to the baseline (mean difference 3.80, 95% CI 2.11–5.49, *p* < 0.005) and placebo (mean difference 4.30, 95% CI 2.45–6.15, *p* < 0.005) groups (Table 5).

## Discussion

The purpose of this study was to evaluate the impact of probiotics on age-related cognitive and emotional decline in a healthy older population. The findings of the present work show that the consumption of *Lactobacillus rhamnosus* and *Bifidobacterium lactis* has a positive impact on mental well-being, leading to improved cognitive function and enhanced emotional state, along with a notable decrease in depressive symptoms.

Recent research has shown the important mental consequences of probiotic consumption through modulation of the GBA in pathologies such as AD or mild-cognitive impairment [28, 29]. In this regard, probiotic administration improves cognitive function in AD patients by reducing oxidative stress markers such as malondialdehyde and C-reactive protein, as well as improving MMSE scores [16]. Similarly, oral administration of *Lactobacillus plantarum* C29 was found to improve cognitive performance in individuals with mild cognitive impairment and this improvement is related to increased serum levels of brain-derived neurotrophic factor (BDNF). The BDNF protein, essential for preserving synaptic function, has been found to be reduced in individuals suffering from cognitive decline and AD, which contributes to the progression of these conditions [21]. Studies focusing on the role of probiotics *Lactobacillus rhamnosus* and *Bifidobacterium lactis* in neurodegenerative diseases and cognitive impairment are limited and mostly come from animal research [30–33]. The beneficial effect of these probiotic strains in neurological pathology could be attributed to their influence on various neuronal processes such as neurotransmitter release, neurogenesis, neuropeptide expression, synaptic plasticity, and neuroinflammation [34–36]. Thus, the administration of probiotics such as *Bifidobacterium lactis* reduces the amount of cerebral amyloid plaques, inflammation, and markers of oxidative stress in rats with AD [37]. Furthermore, it has been shown that the increase in levels of acetylcholine and neurotrophic factors in the brain represents an important mechanism of action of probiotics through the GBA [18, 38].

Cognitive decline associated with ageing is represented as a consequence of neuronal deterioration at the structural and functional level. Multiple neurological changes associated with age-related cognitive decline have been described, such as loss of neural circuits, reduced synaptic plasticity, alterations in the grey and white matter of the brain, demyelination, and damage to the neurovascular unit [39–41]. On the other hand, affective disorders frequently diagnosed in the elderly, such as anxiety or depression, may accelerate cognitive decline and aggravate the disease [42, 43]. Several mechanisms have been investigated to explain the influence of emotion on cognition, including vascular impairment, the hypothalamic–pituitary–adrenal (HPA) axis, inflammatory processes, and changes in neurotrophin levels [44, 45]. Although it is well known that all these cognitive changes associated with the ageing process pose a threat to the physical and mental well-being of the population by creating a state of vulnerability for the development of neurodegenerative diseases and affective problems, nutritional interventions to prevent or delay age-related cognitive decline have not yet been sufficiently explored. A limited number of clinical studies have sought to understand the efficacy of probiotics in cognitive alterations [46, 47]. Several studies have shown improved cognitive function in healthy middle-aged adults up to and including 75 years of age after ingestion of a *Lactobacillus helveticus* fermented milk drink for 8–12 weeks [47, 48]. Recently, another work found improvements in mental flexibility and stress in the PR group compared to the PL group after ingestion of a probiotic containing *Bifidobacterium bifidum* BGN4 and *Bifidobacterium longum* BORI for 12 weeks in elderly people [46]. Regarding studies conducted with the probiotic strains used in our clinical trial, the administration of *Lactobacillus rhamnosus* or *Bifidobacterium lactis* has not shown conclusive results in emotional and cognitive states [49–52].

In our clinical trial, the significant improvement in cognitive function following multispecies probiotic administration experienced by participants may be explained by the existence of several gut-brain interaction pathways [53]. The bidirectional communication is established between the intestinal microbiota and the nervous system by endocrine, immune, and neurological pathways [54]. In the immune pathway, restoration of GM composition through probiotic consumption strengthens the intestinal barrier by preventing bacterial translocation, elevated levels of inflammatory cytokines, and activation of microglia involved in the development of neuroinflammation [55]. GM modulation is also associated with changes in cortisol synthesis through regulation of the HPA axis. In this vein, the results of the present study show an improvement of depressive symptoms in older adults after probiotic treatment. Several studies have demonstrated the ability of administering probiotic strains of the genus *Lactobacillus* to reduce salivary and urinary cortisol levels in subjects under stress and patients with major depressive disorder [56, 57]. In terms of sleep quality, our research suggests that administering probiotics enhances it, as assessed by the PSQI. Although the exploration of the connection between probiotics, sleep quality, and dementia is in its initial phases, early results propose that bolstering gut health through probiotic supplements might offer advantages for sleep quality improvement and the prevention of neurodegenerative conditions or mental alterations [58, 59]. Nonetheless, additional controlled clinical investigations are required to gain a deeper understanding of the underlying mechanisms and to validate these potential benefits.

### Limitations

The present study is not without limitations. Firstly, the research does not incorporate objective measures relating to the characteristics of GM or direct neurological, endocrine, or immunological parameters in older adults, which would be a crucial element to support the role of GBA. Therefore, future research should incorporate physiological measures such as stool bacterial determination, serum concentrations of BDNF, cortisol, indices of inflammation, and oxidative stress. Secondly, this research did not include a thorough control of probiotic strains that could be ingested through other foods that could interfere with the results analyzed. Furthermore, although our results show a benefit of 10-week duration of probiotic intervention, further studies with a longer intervention period or larger sample size are required for some of the measures.

Finally, a second baseline assessment was not conducted after the 4-week washout period. The tools employed, including the MMSE, BDI, and STAI, were deemed highly reliable and stable without intervention, and therefore, an additional baseline assessment was contemplated unnecessary as it would not provide significant new insights. Additionally, from a logistical standpoint, incorporating another baseline assessment would have placed a greater burden on participants, potentially affecting their retention and adherence to the study. We were concerned that the extra testing might discourage continued participation and compromise the reliability of the data collected.

## Data Availability

Data are available upon request to the corresponding author.

## Declarations

### Disclaimer

The data has not been previously presented orally or by poster at scientific meetings.
